# Psychological care for Huntington's disease: A qualitative study exploring interventions in the Netherlands

**DOI:** 10.1111/papt.70034

**Published:** 2025-12-23

**Authors:** Kasper F. Van der Zwaan, Laura C. M. Kuijper, Pearl J. C. van Lonkhuizen, Raymund A. C. Roos, Susanne T. de Bot

**Affiliations:** ^1^ Department of Neurology Leiden University Medical Centre Leiden the Netherlands; ^2^ Department of Public Health and Primary Care Leiden University Medical Centre Leiden the Netherlands; ^3^ Huntington Expertise Center Topaz Overduin Katwijk the Netherlands

**Keywords:** Huntington's disease, multidisciplinary care, psychological intervention, psychotherapy, qualitative research

## Abstract

**Objectives:**

Huntington's disease (HD) is a hereditary neurodegenerative disorder characterized by a range of motor, cognitive, and psychiatric symptoms. Psychological symptoms can arise from being at risk for the disease and from its manifestation, necessitating psychological interventions to address the evolving burden, even before onset. This qualitative study explores the psychological interventions used for HD within the Dutch health care context and identifies barriers and facilitators for their implementation across the different HD disease stages.

**Methods:**

A qualitative approach using semi‐structured interviews was employed, involving 13 experienced psychologists from HD health care facilities in the Netherlands. Thematic analysis was conducted to explore the types of psychological interventions used and the facilitators and barriers affecting their implementation.

**Results:**

The study identified a range of generic and more specific interventions, with their application varying according to the stages of HD. Early interventions focus on genetic counselling and trauma therapy, while mid to late‐stage care incorporates acceptance and commitment therapy (ACT) and cognitive behavioural therapy (CBT) for managing psychological symptoms. Late‐stage interventions shift to mediated approaches involving family and care teams. Key barriers include cognitive impairments and difficulties in referring patients to other mental health care providers, while key facilitators include multidisciplinary collaboration and preventive interventions.

**Conclusions:**

Psychological interventions for HD in the Netherlands are adapted to the disease progression, emphasizing the need for stage‐specific and personalized care. The findings highlight best‐care practices and the importance of early intervention, multidisciplinary collaboration, and evaluation while identifying areas for further research and improvement in care quality.

## INTRODUCTION

Huntington's disease (HD) is a rare autosomal dominant neurodegenerative disorder caused by an expansion of a cytosine–adenine–guanine (CAG) repeat in the HTT gene (Roos, 2010). The disorder is characterized by motor, cognitive, and psychiatric symptoms that vary in onset and progression among individuals (Roos, 2010). Motor dysfunctions, such as but not limited to chorea and gait disturbances, are a pivotal sign of HD. However, cognitive decline and psychiatric symptoms can occur up to 15 years before the onset of motor symptoms (Bates et al., 2015). Currently, there is no cure for HD; treatments focus on the improvement of quality of life by symptomatic relief, which includes medication, physical therapy, and psychological interventions (Stoker et al., 2022).

Psychiatric manifestations and psychological and behavioural problems are integral to HD. Some of these, such as depression, obsessive‐compulsive behaviours, and psychosis, resemble idiopathic disorders. Others, such as executive dysfunction, apathy, irritability, and perseveration, are more specific to HD. Additionally, patients may experience non‐specific (neuro)psychiatric symptoms such as low mood (temporary feelings of sadness, not necessarily linked to mental illness), sexual dysfunction, and sleep disturbances (Rosenblatt, 2007). The aforementioned psychological problems can occur at any stage of the disease. Generally, the following disease stages can be distinguished: At‐risk individuals have a family history of HD but have not undergone genetic testing; pre‐manifest individuals have tested positive for the expanded CAG repeat but show no symptoms. The early manifest stage includes those who are primarily functioning well with mild symptoms; the mid‐stage involves individuals needing assistance with tasks such as work, driving, or managing finances; and the late stage refers to those who are entirely dependent in their daily lives (Shoulson, 1981; Shoulson & Fahn, 1979).

While numerous psychological interventions exist, ranging from waves of behavioural and cognitive therapy to relaxation exercises, their application is not explicitly designed for HD (Hayes & Hofmann, 2021; World Health Organization, 2024). Recent scoping reviews on the effectiveness of psychological interventions have been conducted for other neurological diseases like Parkinson's disease and multiple sclerosis (Fiest et al., 2016; Zarotti et al., 2021). These reviews primarily focus on establishing evidence‐based interventions but do not provide a comprehensive overview of the interventions implemented in clinical practice settings.

There is also limited research on evidence‐based psychological interventions in the context of HD, and the availability and use of these interventions can vary significantly depending on the country or health care context. Different health care systems may have distinct approaches to providing and implementing psychological interventions, leading to variations in access and use (Simpson et al., 2021). Experts have pointed out that, despite the lack of strong evidence‐based studies for HD, clinical experience suggests that psychological interventions can be beneficial in managing neuropsychiatric symptoms (Anderson et al., 2018). Moreover, a recent call was made for a detailed overview of evidence‐based and best‐practice psychological interventions in HD (Zarotti et al., 2022). Given the complexity and variability of HD, it is important to understand which psychological interventions are currently employed in practice, when they are applied, and how they are adapted to patient needs within a specific health care context. The current qualitative study, therefore, addresses this knowledge gap by providing an in‐depth look at the psychological interventions used for HD by specialized HD psychologists in the Netherlands, and how they are implemented at different stages of the disease.

## METHODS

### Design and participants

This qualitative study explores the psychological interventions used in the Netherlands for all stages of HD using thematic analysis (Braun & Clarke, 2006).

**FIGURE 1:**
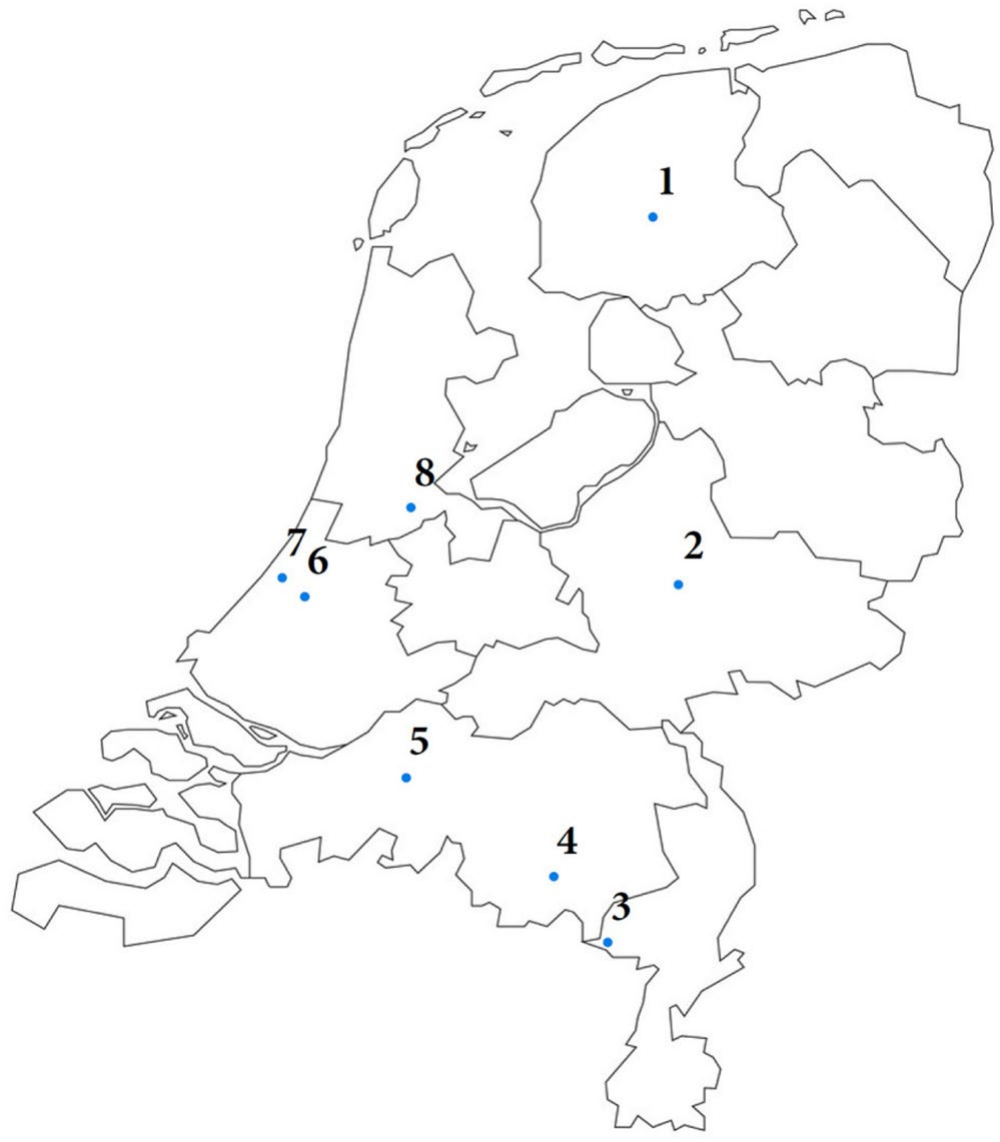
Locations of care facilities where psychologists were employed. 1 = Grou: Zorgcentrum Noorderbreedte, 2 = Apeldoorn: Atlant, locatie Heemhof, 3 = Weert: Zorgcentrum Land van Horne, 4 = Eindhoven: Zorgcentrum Archipel locatie Landrijt, 5 = Raamsdonksveer: Mijzo, Zorcomplex de Kloosterhoeve, 6 = Leiden: Leids Universitair Medisch Centrum dept. Clinical Genetics, 7 = Katwijk: Zorgcentrum Topaz Overduin, 8 = Amsterdam: Amstelring, locatie Willem Drees Oostpoort.

Psychologists were invited, either via email or in person during a yearly meeting of the *Huntington KennisNet Nederland* (HKNN; the Dutch Huntington's Disease knowledge network), to participate. They were drawn from the eight specialized (chronic) care facilities providing psychological care for HD across the Netherlands (Figure 1). Participants had to have at least 2 years of experience with HD.

In recruiting participants, we aimed for a balanced representation of care settings, including psychologists involved in inpatient and outpatient care. The study adhered to ethical standards for confidentiality and professional conduct, with participation being voluntary and informed consent implied through participation. Formal ethical approval was not required under Dutch law (Medical Research Involving Human Subjects Act, WMO), as this study involved interviews with health care professionals and did not include patients or interventions. The study was also conducted in accordance with the Declaration of Helsinki regarding ethical principles for medical research involving humans, including respect for confidentiality and the protection of participants' rights.

### Data collection

Interviews were conducted between May 23, 2023, and December 11, 2023. All interviews were conducted in Dutch by one researcher (KZ), and all were held in person except for one interview; this interview was repeated online due to a prior malfunction of the recording device. The total duration of interviews was 14 h and 5 min, with a mean duration of 1 h and 5 min per interview. All interviews were recorded on a laptop and transcribed intelligent verbatim by a professional transcribing agency.

The semi‐structured interview guide (see Appendix A), designed by KZ with assistance from an experienced psychologist in the HD field, included topics on the demographic and professional background of the psychologists, their understanding of the term ‘psychological interventions’, their global and Huntington‐specific experience, the different stages of HD they encounter, types of care‐related questions and specific problems faced by patients, psychological interventions used and their perceived effectiveness, barriers, and challenges in implementing interventions and interventions not yet used but desired by the professionals. Participants’ input directed the future course of the interviews.

### Data analysis

Data analysis was performed using Atlas.ti version 24.1.1.30813 (ATLAS.ti Scientific Software Development GmbH, 2024). Thematic analysis was conducted to investigate the psychological interventions currently implemented in Dutch HD care, as well as the factors that facilitate or hinder the provision of psychological care (Braun & Clarke, 2006). To ensure consistency across all interviews, the interviewer provided a balanced, predefined definition of a psychological intervention after participants shared their own definitions. Psychological interventions were likened to tools (e.g., a hammer or screwdriver) that can be used in different construction tasks (therapeutic approaches), illustrating how certain techniques can be applied across various therapeutic contexts. This analogy helped align perspectives by encompassing both broad and narrow interpretations of psychological interventions, clarifying what constitutes an intervention and what does not.

The analysis consisted of the following steps: (1) To gain familiarity with the data, all transcripts were read by the first (KZ) and second author (LK); (2) Both authors then deducted an initial code scheme based on anticipated and emergent themes from the interviews (top‐down); (3) Both researchers coded each interview; during coding, codes were inductively and iteratively updated and refined after each interview (bottom‐up); (4) Coding discrepancies were addressed through regular discussions and consensus‐building between KZ and LK or by consultation with the third author PL; and (5) Codes were organized into themes reflecting interview patterns and insights.

To explore barriers and facilitators to implementing psychological interventions at different stages of HD, we partially followed the Consolidated Framework for Implementation Research (CFIR) (Damschroder et al., 2022). CFIR helped to structure the thematic analysis further. It resulted in a list of recommendations that leverage the strengths of facilitators and address the barriers.

Transcripts were analysed in the original language to minimize interpretative distortion. For publication, selected illustrative quotations were translated into English by the first author and reviewed by a co‐author to ensure accuracy, clarity, and preservation of meaning. Minor linguistic adjustments were made to improve readability while maintaining the original intent and tone.

## RESULTS

### Participants' characteristics

Thirteen psychologists (11 female) participated in the study. All were HKNN members and had at least 4 years of experience with HD, with an average of 14.8 years (range: 4–23 years). Table 1 provides a per‐participant overview of demographic and professional characteristics. Three themes were identified across all interviews: (1) Understanding the scope of psychological interventions, (2) Psychological interventions across stages of HD, and (3) Challenges and facilitators in delivering psychological care for HD.

**TABLE 1:** Demographic and professional characteristics of the participating psychologists.

Psychologist	Gender	Years of experience	Years of experience HD	Inpatient or outpatient	Professional background	Stages
A	F	22	15	Both	Licensed Health Care Psychologist	All stages
B	F	20	5	Both	Licensed Psychotherapist (CBT and EMDR)	All stages
C	F	14	12	Both	Licensed Health Care Psychologist	All stages
D	F	4	4	Both, more inpatient	Licensed Health Care Psychologist	All stages
E	M	13	9	Both, more outpatient	Unlicensed Psychologist	All stages
F	F	20	20	Outpatient	Licensed Health Care Psychologist	All stages focus on at‐risk and pre
G	F	19	7	Both	Licensed Health Care Psychologist	All stages
H	F	10	5	Outpatient	Unlicensed Psychologist	All stages focus on pre‐ to mid‐stage
I	F	8	4.5	Both	Licensed Health Care Psychologist	All stages
J	M	11	7	Both, more outpatient	Unlicensed Psychologist	Pre‐manifest to mid‐stage
K	F	9	6	Both	Health Care Psychologist in training	All stages
L	F	20	20	Both	Licensed Health Care Psychologist	All stages
M	F	23	23	Both	Licensed Clinical Neuropsychologist PhD	All stages

*Note:* In the Netherlands, only BIG‐registered psychologists (i.c., GZ‐psychologist) are licensed to practice independently. MSc psychologists are unlicensed and typically work under the supervision of a licensed psychologist.

### Theme 1: Understanding the scope of psychological interventions

Participants interpreted psychological interventions in either a broad or narrow sense. Six of the 13 psychologists defined psychological interventions broadly, while three adopted a narrower perspective. Four psychologists expressed both broad and narrow definitions, indicating a more nuanced understanding of the construct. Broadly, interventions are seen as any psychologist's action, formal, or informal (e.g., joining a patient for coffee), contributing to a client's positive change. The goal is to enhance the client's well‐being, regardless of the method used:

I believe that essentially, everything I'm involved in could be considered a psychological intervention. Even a casual conversation with a family member in the hallway can involve therapeutic elements due to my professional background. As a psychologist, I believe that every interaction is, in a sense, both a diagnosis and a treatment. (M)

Narrowly, interventions are viewed as structured, protocol‐based treatments designed to address specific psychological issues systematically:

If you look at it technically, a psychological intervention is essentially any protocol‐based treatment. (K)

The preventive function of interventions was frequently mentioned as the counterpart to narrowly defined interventions that focus solely on treating DSM diagnoses. In practice, much of the work of the psychologists focuses on addressing symptoms before they fully develop. Patients often visit health care facilities in the early or pre‐manifest stages of the disease and receive annual follow‐ups. As a psychologist noted, this enables a deep understanding of patients, not only in terms of their illness, but also on a more existential level. An example of preventive psychological intervention is treating low mood or guiding for managing stressful situations, which can help to prevent the onset of conditions like depression and anxiety.

Imagine being in the premanifest stage and quite passive. We need to teach you new skills, which could be beneficial later. This is also psychotherapy and offers intervening opportunities, these psycho‐preventive measures [sic] See, we just invented a new research term. (K)

Some participants noted some generic interventions or approaches, although less frequently mentioned, addressing specific challenges in HD. For instance, cognitive revalidation was reported to assist with early cognitive and work‐related difficulties. The directive empathetic approach was highlighted as particularly useful for managing anosognosia and supporting patients from lower socioeconomic backgrounds. Group therapies provided valuable communal support, including psychoeducation, peer support, emotion‐focused therapy (EFT), and group relaxation techniques. Solution‐focused therapy was noted for its effectiveness in managing grief, loss, and fostering problem solving skills that can address disease progression, while schema therapy was recognized for targeting Axis II personality disorders. Additionally, attachment focused relationship therapies, such as Hold Me Tight (Johnson, 2011; Petzke et al., 2022) (a form of EFT), were mentioned as beneficial for maintaining and improving relationship dynamics as the disease progresses.

We've got the new ‘Hold Me Tight’ training. A colleague has been trained in it and will train us, so I hope we can help people early on when they face relationship issues at home. However, we worry we might see them when it [HD] is already too advanced, as they still need to do homework for it. We're starting with that, which I find really exciting. (D)

In terms of specific interventions and approaches mentioned, the psychologists highlighted several methodologies, including acceptance and commitment therapy (ACT), cognitive behavioural therapy (CBT), and eye movement desensitization and reprocessing (EMDR). The choice of intervention varies significantly across different stages of HD, reflecting the specific psychological needs and challenges faced by patients as the disease progresses. We will explore this further in the second theme.

### Theme 2: Psychological interventions across stages of HD

**FIGURE 2:**
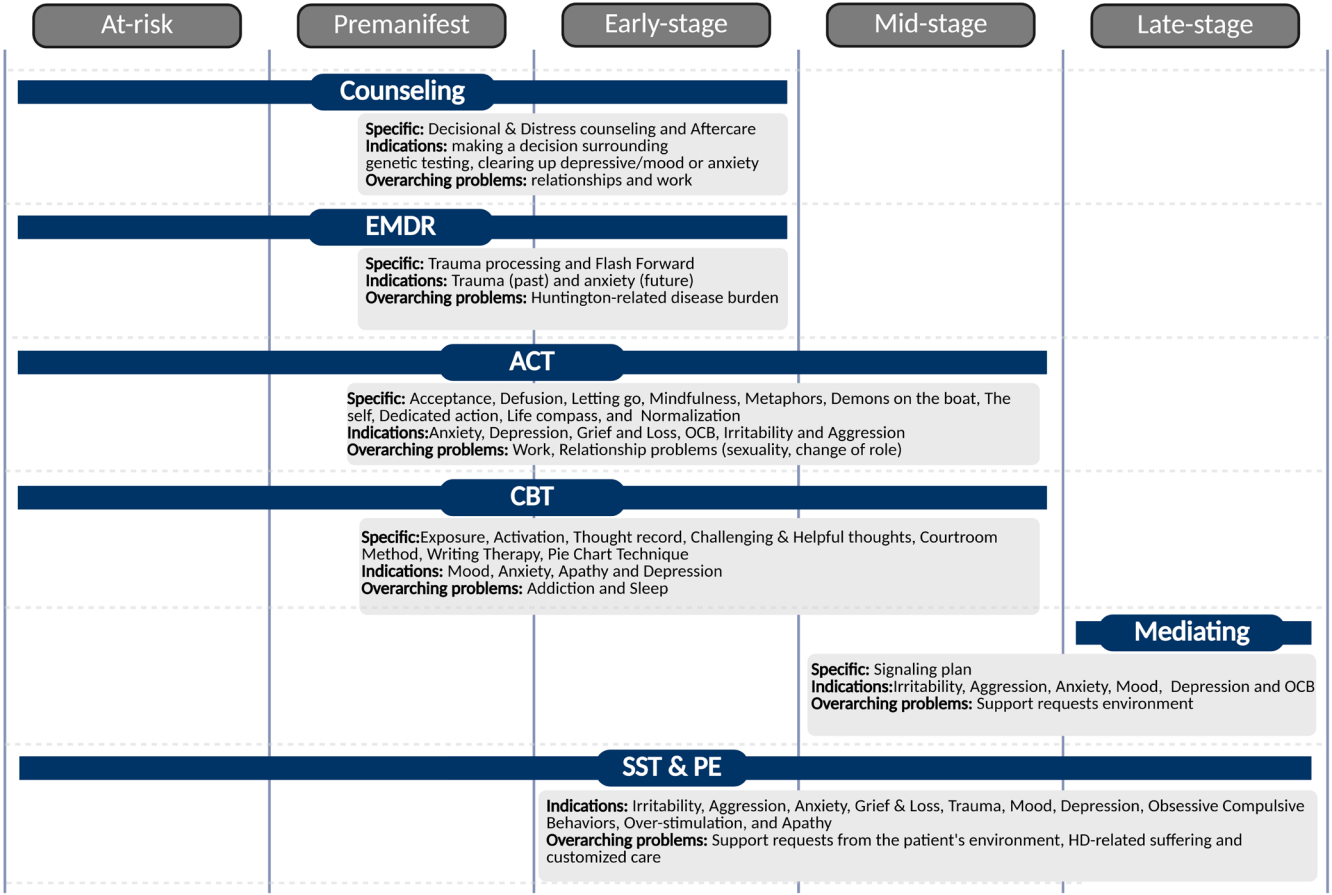
An overview of the use of and psychological interventions employed in Dutch HD care. ACT, acceptance and commitment therapy; CBT, cognitive behavioural therapy; EMDR, eye movement desensitization and reprocessing; HD Care, Huntington's disease care, SST & PE, structuring and supporting conversation techniques and psychoeducation.

A comprehensive overview of the psychological interventions across the several stages employed in Dutch HD care is given in Figure 2 and will be elaborated on in the following paragraphs.

#### At‐risk, pre‐manifest, and early stage

In the initial stages of HD, including at‐risk, pre‐manifest, and early stage, psychologists emphasized the importance of targeted psychological interventions to address emerging (neuro)psychological challenges. According to participants, genetic counselling is a crucial component of early care, with a strong focus on decisional and distress counselling to help individuals manage the emotional and practical implications of genetic testing.

If someone struggles psychologically at the start, we must intervene. Our goal is to help them recover and make informed choices about genetic testing. We'll support them in managing uncertainty, whether they test or not. Ultimately, we aim for their psychological well‐being. (F)

A desire to start a family is a common reason for individuals to consider genetic testing. Psychologists observed that patients who initially seek support during this early stage often do not return for quite some time but later seek outpatient care as symptoms become more noticeable, particularly when early signs of the disease begin to interfere with their ability to work. Therefore, early interventions often focus on stress reduction or mood stabilization in both work‐related and home situations.

They [patients] realize: “I can't do my job anymore. I used to be able to, but now I can't. Is this burnout, or is it early signs of Huntington's?” (F)

Four psychologists also identified eye movement desensitization and reprocessing (EMDR) as a valuable intervention for processing trauma in these early stages. They observed that EMDR helps to reduce the emotional impact of traumatic memories and aids individuals in coping with the psychological burden associated with their HD risk or diagnosis.

One psychologist suggested that treating HD patients with EMDR is more manageable than treating regular trauma patients, as the HD working memory can be more easily and quickly fully taxed, an essential aspect of EMDR therapy. However, its use was reported less frequently in later disease stages.

We've had national discussions about whether EMDR is really that different for Huntington's. … Perhaps the mode of distraction and initial explanation are different. I think you need to evoke more emotion in the moment and use that distraction. (G)

Three psychologists wondered whether flashforward interventions could treat specific disease‐related anticipatory anxieties; only one of the participants had ever used it without a noticeable positive effect.

#### At‐risk to mid‐stage

In the beginning, but especially as HD progresses and symptoms and signs along with functional decline increase, participants reported that acceptance and commitment therapy (ACT) and cognitive behavioural therapy (CBT) have become increasingly relevant. ACT focuses on helping individuals accept difficult emotions and thoughts while committing to actions that are aligned with their values to create a more meaningful life. All psychologists highlighted ACT, commenting that ACT is particularly effective in helping patients manage anxiety, depression, grief, and obsessive‐compulsive behaviours through techniques, such as acceptance, defusion, and mindfulness. Two psychologists mentioned the use of ACT in work‐related problems and relationship problems, for example, sexuality and the change of family dynamics.

It's not [only] about accepting, it's about tolerating. And that's also a kind of intervention that has a certain power because people then think: “Oh, yes, that's what I can do”. (F)

The psychologists noted that while ACT encourages patients to embrace their thoughts, CBT focuses more on challenging and altering unhelpful thought patterns. CBT is a widely used form of psychotherapy that focuses on identifying and changing negative thought patterns and behaviours to improve emotional regulation and develop healthier coping strategies. CBT thereby addresses issues related to mood, anxiety, depression, and passivity. Three psychologists underlined the necessity of good cognitive abilities, especially executive functioning, for effective CBT treatment.

Generally, CBT is more suited for people who are cognitively functioning well. So when it comes to pre(motor)manifest and (motor)manifest stages, you can only use CBT if they still have good learning capacity and can complete homework. (G)

#### Late stage

In the late stages of HD, when patients become (entirely) care‐dependent, the six participants working at residential units noted that psychological interventions often shift to mediated interventions due to significant cognitive decline. Mediating therapy treats the patient by treating their environment: the psychologist trains caregivers to modify interactions that shape the patient's behaviour. The psychologists emphasized the importance of mediating therapy, which primarily involves guiding family members and care teams in understanding and managing the patient's complex behaviours and needs rather than directly intervening with the patient. They highlighted the use of tools like signalling plans in nursing homes as essential for managing symptoms such as irritability, aggression, anxiety, mood disorders, depression, and obsessive‐compulsive behaviours. Participants described signalling plans as preventive strategies designed to identify early signs of severe psychological crises and outline steps for timely intervention by the patient, their environment, and professionals to prevent escalation. They employ a wide range of intervention tools to accomplish this.

Mediation therapy is a valuable tool. I often use solution‐focused techniques and CBT, both with individuals and teams. (D)

The focus, they explained, is on providing tailored support to the patient's environment to address the complex needs of late‐stage HD.

#### All stages

All psychologists reported that across all stages of HD, structuring and supportive conversation techniques (SST), along with psychoeducation (PE), are consistently applied as core interventions:

You're offering psychoeducation, and that's really key. People often feel a huge sense of relief when they understand what's happening to them. Someone in this situation might think, ‘I must be going crazy. Everyone else can do this, why can't I?’ It can be very isolating. Psychoeducation helps them understand that they're not alone and that their experiences are common. (F)

These techniques were highlighted as effective in managing symptoms, such as irritability, aggression, anxiety, grief, trauma, and depression, as well as addressing neuropsychological issues like obsessive‐compulsive behaviours, overstimulation, and passivity. According to the psychologists, SST and PE serve as preventive measures to address emerging complaints, and they are also essential therapeutic tools, providing validation and helping individuals feel heard, understood, and not alone in their experiences. Additionally, the psychologists emphasized the importance of these techniques in providing crucial support for caregivers, enabling them to tailor care to the patient's evolving needs.

Sometimes I teach about Huntington and its effects on the brain in the HD cafe. We also consult and educate other institutions when they call with questions. (D)

#### Switching from technique

Psychologists have described that transitions between therapeutic techniques are rarely abrupt but often emerge from a gradual shift in cognitive, emotional, or contextual factors. Key triggers for changing interventions included cognitive decline, particularly in executive functioning, reduced patient insight or motivation, increased behavioural symptoms, or shifting therapeutic goals. For example, when patients in the early stages of HD initially benefited from CBT but later experienced difficulties with abstract reasoning or homework, psychologists reported transitioning to more experiential approaches, such as ACT, or to supportive strategies that required less cognitive effort. In more advanced stages, this process continued towards mediated therapy or caregiver‐focused interventions. Rather than following a predefined protocol, these transitions were guided by ongoing clinical assessment and the psychologist's familiarity with the patient's evolving capabilities and needs. A consolidated overview of the psychological interventions mentioned throughout this theme, including their theoretical underpinnings, typical application stages based on our findings, and practical considerations in the context of HD care, is provided in Table 2.

**TABLE 2:** Theoretical basis and practical considerations of interventions used in HD.

Intervention	Theoretical basis	When applied	Practical considerations
Cognitive behavioural therapy (CBT)	Challenging and restructuring dysfunctional thoughts and behaviours (Fenn & Byrne, 2013).	Pre‐manifest and early manifest stages with intact cognition. For depression, anxiety, OCD	Requires executive functioning; homework must be adapted; effectiveness decreases with cognitive decline
Acceptance and commitment therapy (ACT) (Hayes, 2004; Hayes & Hofmann, 2021)	ACT uses acceptance, mindfulness, and value‐based action to increase psychological flexibility (Hayes & Hofmann, 2021)	All stages, mainly early manifest. For coping with progression, loss, and uncertainty	More flexible than CBT; metaphors may need simplification; client motivation required
Psychoeducation	Providing information about the disease, symptoms, and consequences (Sarkhel et al., 2020)	All stages, including family; important for grief and misunderstandings	Powerful for normalization; needs repetition; adapt to cognitive decline
Mediating therapy	Indirect therapy focused on caregivers; based on behavioural principles	Mid to late stages; for behavioural issues and reduced communication	Depends on care team involvement; psychologist coaches and challenges the team
Supportive and structuring conversation techniques	Safe space for expression, structuring, and validation	All stages; especially when no concrete treatment goal is present	Essential but underestimated; requires emotional presence with patient despair
Eye movement desensitization and reprocessing	Processing of dysfunctionally stored memories through bilateral stimulation (Oren & Solomon, 2012)	Pre‐manifest and early stages; used for trauma, nightmares, and anticipatory anxiety	Challenging with cognitive impairments; protocols often need adaptation

*Note:* This table synthesizes information derived primarily from the interview material, complemented by theoretical references where available. Evidence‐based interventions (e.g., CBT, ACT, EMDR, psychoeducation) are supported by established literature, whereas mediating therapy and supportive/structuring conversation techniques reflect approaches that emerged strongly from the participants' professional practice within the Dutch healthcare context and are consistent with national clinical guidance on psychological care in long‐term and nursing home settings (e.g., Allewijn & Haaring, 2004; Klaver, 2006; Klaver & A‐Tjak, 2006).

### Theme 3: Barriers and facilitators in delivering psychological care for HD

#### Barriers

A central barrier in HD psychological care lies in the progressive cognitive decline, as it limits the effectiveness of interventions like cognitive behavioural therapy (CBT) and acceptance and commitment therapy (ACT), which rely on planning, homework, and metacognitive abilities (i.e., thinking about thinking). All psychologists underlined the issues of cognitive decline and its limitation of psychological interventions. However, one psychologist reflected that they also sometimes use it as a helpful starting point.

Yes, where cognition is often an easy entry point initially to connect with people. Especially when individuals are experiencing cognitive impairments or are concerned about them, you can see that there's motivation to gain control over it, to understand it, and to manage it better for themselves. (M)

Yet, as the cognitive problems progress, patients' impairments like anosognosia can emerge. The lack of disease awareness in anosognosia renders patients unable to recognize their limitations, further complicating therapy that relies on introspection and goal‐setting.

If people truly have no insight, if their awareness of the disease is completely absent—if they even say there's nothing wrong—there's very little you can do [directly] (J)

Closely related to anosognosia is avoidant coping, in which, patients may resist interventions, avoid care providers, or dismiss the severity of their symptoms. Topics that patients often avoid are the impact of HD on driving ability and advanced life planning. One‐third of the psychologists mentioned that, such avoidant behaviours often leave them in a difficult position, as they must navigate between respecting a patient's autonomy and intervening to prevent further psychological decompensation.

Another significant barrier is the uncertainty regarding the effectiveness of psychological intervention in HD. Without standardized therapeutic measures, clinicians often depend on patient feedback as the primary indicator of success. While valuable, this feedback lacks the robustness required for definitive evidence of treatment efficacy. Furthermore, the absence of tailored, evidence‐based protocols leaves clinicians in uncharted territory, relying on improvisation or adaptation from general approaches — which makes psychologists feel like they are just improvising. Additionally, some psychologists struggle to determine when to discontinue therapy, often continuing for years without a clear goal or measurable outcomes.

Four psychologists mentioned that referrals of HD patients to other (specialized) mental health care providers (HCPs) are sometimes unavoidable, particularly when specific expertise is required for issues beyond the scope of HD‐focused care, such as trauma processing or treatment of other comorbid mental health conditions. These referrals can pose challenges, as non‐HD‐specific providers may not be familiar with the nuances of HD and often misattribute idiopathic symptoms solely to the disease. To address this gap, the involvement of HD psychologists as consultants was identified as a potential solution. For an overview of the barriers (and facilitators) see Table 2.

#### Facilitators

Despite the significant barriers, several facilitators can enhance the delivery of psychological care for HD patients. Overall, being flexible and creative in tailoring interventions to the specific cognitive and psychological capacities of patients improves HD psychological care, especially when using (parts of) protocolized interventions not specifically designed for HD.

The aforementioned avoidant coping can be managed by building early relationships with patients and getting to know them on an existential level, allowing for a better understanding of their values and motivations. Also, assertive outreach care was highlighted by two psychologists as crucial for patients who resist treatment. Assertive outreach is a proactive approach to care that involves persistent engagement and support for individuals who resist treatment, often through home visits.

We often engage in what might be called assertive outreach care. People may not want us involved, but we strive to maintain contact. Sometimes, patients deny their illness. In such cases, we're just glad if they [eventually] come here and have a positive view of us. Building that trust can take years, but we hope that when they eventually need care, we can be there for them, especially if their situation becomes dangerous or they start neglecting themselves. (I)

Not only focusing on the patient but also relying on system‐oriented care (e.g., care takers and family members) plays a crucial role in addressing avoidant coping. This approach was particularly emphasized by psychologists providing extramural care, as opposed to mediation therapy in advanced stages, which primarily supports care teams and families in managing complex late‐stage behaviours.

Furthermore, the focus on preventive work, which emphasizes addressing symptoms before they escalated, was highlighted as an important facilitator of psychological care. By conducting annual follow‐ups and engaging patients during the early or pre‐manifest stages of HD, HCP can develop a deeper understanding of their patients, both clinically and existentially. This proactive approach helps identify early signs of distress and equips patients with essential coping skills, mitigating the risk of developing more severe conditions such as depression and anxiety.

Another facilitator is the multidisciplinary teams in Dutch nursing home care, which include specialists in elderly care medicine, physiotherapists, occupational therapists, speech therapists, dieticians, social workers, and nurses who are also specialized in HD care. The multidisciplinary approach enhances holistic management by addressing the diverse medical, psychological, and social needs of patients. Six psychologists highlight the benefits of this integrated care; however, they also note that coordinating efforts across disciplines can be time‐intensive and complex.

The nursing home care is further supported by Neurologists, Psychiatrists and Clinical Genetics departments that are specialized in HD care. The well‐organized referral structures established among these HCP streamline the process of connecting patients to the appropriate resources, ultimately improving treatment outcomes. For an overview of the facilitators (and barriers) see Table 3.

**TABLE 3:** List of barriers and facilitators of psychological interventions and care.

Barriers	Details
Cognitive decline	Limits effectiveness of CBT and ACT due to reliance on planning and metacognitive abilities Initial cognitive awareness may create a window for engagement but might diminish over time
Anosognosia and avoidant coping	Patients that lack disease awareness, which complicates therapy reliant on introspection Avoidant coping behaviours include resisting interventions, avoiding care providers, and minimizing symptoms
Uncertainty of effectiveness	Lack of standardized therapeutic measures; patient feedback is primary but insufficient indicator of success Absence of tailored, evidence‐based protocols forces reliance on improvisation
Unclear Termination	Therapies can continue for years without clear goals or outcomes
Referrals to non‐HD specialized care	Mental health care providers unfamiliar with HD may misattribute idiopathic symptoms solely to HD. This can result in referrals being declined
Facilitators	
Flexibility and creativity	Tailoring interventions to individual cognitive and psychological capacities enhances outcomes Adapting therapeutic strategies as cognitive impairments progress
Assertive outreach	Proactive approach involving persistent engagement through home visits and continued contact Builds trust with resistant patients, leading to eventual care acceptance
Systemic‐oriented care	Engaging caregivers and family members ensures holistic support Further helps manage avoidant coping by addressing patient concerns in a broader context
Preventive work	Emphasis on early intervention and annual follow‐ups in pre‐manifest stages. Equips patients with coping strategies to mitigate severe conditions like anxiety or depression
Multidisciplinary collaboration	Teams include specialists, such as physiotherapists, dieticians, and social workers addressing diverse needs. Coordinating care across disciplines can be time‐intensive but enhances holistic management
Referrals to specialized HD care	Structured referral systems connect patients with neurologists, psychiatrists, and clinical genetics departments specialized in HD care

## DISCUSSION

HD presents a multifaceted challenge that requires a nuanced and often adaptive approach to care across psychological, cognitive, and physical domains. Our study offers an overview of the psychological interventions applied at various stages of HD within the Dutch care context, emphasizing the critical importance of early and stage‐specific care. Additionally, we explore the barriers and facilitators that impact these interventions. Overall, psychologists working in HD care in the Netherlands often need to be creative and flexible in applying psychological interventions. While this study does not provide formal outcome measures of therapeutic effectiveness, its primary aim was to capture the clinical reasoning and intervention strategies of experienced HD psychologists in real‐world settings. In a field where empirical evidence for psychological interventions in HD is still limited, practice‐based knowledge offers valuable insight into how care is currently delivered and adapted to the needs of patients and families. The reported practices reflect perceived clinical benefit and represent a form of expert consensus on what works in HD psychological care. Future studies are needed to evaluate these interventions systematically, in other cultures and insurance systems, including the perspectives of patients and their families, to determine their effectiveness, relevance, and impact on quality of life.

### Competence without protocols

The complexity and heterogeneity of HD make it challenging to establish guidelines, leading to a reliance on best practices rather than standardized protocols. The cognitive deficits, combined with the psychiatric symptoms and the progressive nature of the disease, are unavoidable, and psychologists must continually adapt to these challenges. This uncertainty about the effectiveness of their interventions left many psychologists in the present study questioning the structured nature of their approaches, describing their work as lacking a clear framework despite being grounded in professional expertise.

This is reflected in a relatively broad perspective of what a psychological intervention can encompass, discussed in our first theme. Despite this perceived lack of structure, our findings reveal a surprisingly coherent treatment strategy that has developed over years of clinical experience. Psychologists consistently use interventions drawn from acceptance and commitment therapy (ACT), cognitive behavioural therapy (CBT), and mediation techniques, adapting them to different stages of the disease. This consistency in practice suggests that while formal evidence may be lacking, years of practical experience have resulted in an aligned approach to care.

Nevertheless, the absence of clear international guidelines in psychological care for HD highlights the need for research to validate and standardize these practices, providing evidence‐based support and enhancing practitioners' confidence in delivering psychological care for HD: an important topic in theme three.

In multiple sclerosis (MS), for instance, randomized controlled trials (RCTs) have been instrumental in validating tailored psychological interventions for addressing cognitive deficits, fatigue, and depression (Thomas et al., 2006). The advancements in MS care underscore the potential benefits of adopting similar validated protocols for HD, providing practitioners with evidence‐based tools to navigate the complexities of the disease. This also relates to our third theme, which emphasizes the need for systematic assessments and targeted training to bolster practitioners' sense of competence.

Systematically assessing the effectiveness of interventions through regular measurements combined with targeted training on Huntington‐related topics can also contribute to the sense of competence.

Another essential clinical consideration highlighted in this study is the need to adapt therapeutic approaches in response to disease progression, particularly cognitive decline. As HD advances, patients gradually lose the cognitive flexibility, insight, and memory capacities required for interventions such as CBT. Psychologists in this study reported that they often transitioned from cognitively demanding therapies to approaches that are less dependent on abstract reasoning, such as acceptance and commitment therapy, supportive conversations, and ultimately, caregiver‐mediated strategies. These transitions are rarely standardized or protocol‐driven, but instead reflect adaptive clinical reasoning in response to the evolving needs of patients. This underscores the importance of stage‐specific care and the need for further research into how psychological interventions can be dynamically tailored throughout the disease course.

### Influence of the Dutch HD care landscape

The Dutch HD care landscape has strengths and limitations. Psychologists collaborate closely with neurologists, psychiatrists, physiotherapists, social workers, and genetic counsellors, providing a strong foundation for personalized, stage‐specific interventions. This integrated system allows for various psychological interventions across all stages of the disease, from providing genetic counselling and emotional support in early stages to guiding caregivers and managing complex behaviours in later stages. Previous studies in the Netherlands have highlighted the significance of this established multidisciplinary approach (Veenhuizen & Tibben, 2009). However, referrals to non‐HD‐specific mental HCPs, who may have more experience treating specific mental disorders, can sometimes present a challenge. For example, HD psychologists in HD centres are not always equipped to handle issues such as trauma processing, while HD patients may very well develop post‐traumatic stress disorder (PTSD) from childhood experiences. In these cases, it is essential to refer patients to specialized trauma clinics for appropriate care. However, as psychologists noted, these specialized HCPs often refer patients back, citing the prominence of HD as a complicating factor that they feel unprepared to address. This misconception creates additional challenges and delays in care. To address this, participants recommended that HD psychologists act as consultants for other mental HCPs in treating HD patients for non‐HD mental disorders. This approach would bridge the knowledge gap and ensure that HD patients receive appropriate psychological support outside of specialized settings. Furthermore, the financial structure in the Netherlands, where a clinical HD diagnosis covers most outpatient psychological care, further supports personalized care efforts.

### Study's strengths and limitations

The strengths of this study are its in‐depth look at psychological interventions throughout different stages of HD and its identification of both commonly and less frequently used approaches. It provides valuable insights into psychologists' practical experiences and their different strategies, including barriers and facilitators reported directly from the professional field. However, there are notable limitations. The reliance on self‐reported data from a relatively small sample may limit the generalizability of the findings. However, we have ensured comprehensive coverage and avoided data saturation by including inpatient and outpatient psychologists from the same facility when applicable. This approach prevented duplicate information and ensured a balanced view of care practices. In addition, we have covered most, if not all, care facilities providing specialized psychological HD care in the Netherlands. The study's limited geographical scope may restrict the applicability of its results to other countries with different health care systems.

### Future research directions

Future research should address several key areas to advance our understanding of psychological interventions for HD. First and foremost, developing evidence‐based protocols to optimize HD care and enhance psychologists' confidence is essential. Similar studies as the current study in the Netherlands should be conducted in different countries to develop a more comprehensive understanding. Moreover, it will help identify variations and commonalities in barriers, facilitators, and psychological interventions across diverse health care systems. Additionally, evaluating the long‐term effectiveness of various psychological interventions, including less commonly used therapies like EMDR‐based Flashforwards for anticipatory anxiety, is crucial. Exploring systemic and contextual therapies and expanding research into caregiver support and family dynamics will provide deeper insights into optimizing HD care and strengthening evidence‐based practices.

### Practical recommendations

First, it is essential to continue and expand the mapping of psychological interventions to ensure effective, stage‐specific care tailored to patient needs. Replication of this study in other countries should reveal differences and similarities in the barriers, facilitators, and psychological interventions used in HD care, further deepening our understanding and strengthening the evidence for best practices and international guidelines.

To better manage HD, training programmes should focus specifically on the psychological aspects of the condition for HD specialized psychologists. Enhancing their expertise in areas such as trauma therapy and complex behavioural management will equip them with the necessary skills to address the unique psychological challenges of HD, increasing their competence and confidence. Improved training will lead to more effective care and better patient outcomes. Additionally, expanding support programmes for caregivers and family members is crucial for helping them manage the psychological and practical challenges associated with HD. Strengthening these support systems will reduce resistance to care and improve the overall caregiving environment, benefiting patients and their families.

## CONCLUSION

This study, based on the experiences of specialized HD psychologists, underscores the importance of stage‐specific and personalized psychological care for HD. It highlights commonly used practices such as early intervention, multidisciplinary collaboration, and tailored psychotherapeutic interventions. Despite the lack of formal guidelines, integrating various psychological strategies driven by clinical experience can benefit managing HD, according to experienced HD psychologists. In addition, the study identifies key barriers and facilitators that influence the use of psychological interventions in HD. Future research should focus on further validating these practices and their effectiveness and developing standardized guidelines to improve care globally. Expanding studies to different health care systems and evaluating emerging therapies will further enhance our understanding and effectiveness of psychological interventions in HD.

## AUTHOR CONTRIBUTIONS

**Kasper. F. Van der Zwaan:** Conceptualization; investigation; methodology; writing – original draft; visualization; writing – review and editing; formal analysis; data curation. **Laura C. M. Kuijper:** Conceptualization; investigation; data curation; formal analysis; writing – review and editing; methodology. **Pearl. J. C. van Lonkhuizen:** Methodology; writing – review and editing; supervision. **Raymund. A. C. Roos:** Supervision; writing – review and editing. **Susanne. T. de Bot:** Writing – review and editing; supervision.

## FUNDING INFORMATION

This work was supported by the LUMC and the generous contribution from the Bontius stichting, generated by private donations.

## CONFLICT OF INTEREST STATEMENT

The authors declare no conflicts of interest.

## Data Availability

The data that support the findings of this study are available from the corresponding author upon reasonable request.

## APPENDIX A SEMI‐STRUCTURED INTERVIEW

Good morning, and thank you for taking the time to meet with me today. I am researching psychological interventions used by psychologists who provide care to people with Huntington's disease and/or their family members. Through this interview, I hope to learn more about the psychological interventions you use in your practice.

I would like to record our conversation to focus entirely on your responses if that is all right with you. The interview will be transcribed later, and all references or names that could identify you will be removed to ensure complete anonymity. All information you provide will be treated as confidential and anonymous and used solely for scientific research. The researchers will only access the recordings and transcripts in this study.

[start recording]

Could you tell me about your background and experience working with people with Huntington's disease?

- **Prompt questions:**
  ○ What exactly is your background? For example, are you a general psychologist, health care psychologist, psychotherapist, clinical neuropsychologist, or clinical psychologist?
  ○ What type of institution do you work in? For instance, do you work in a nursing home, a mental health care institution, an academic hospital, a general hospital, or a clinic?
  ○ How long have you been working there?
  ○ Is the institution/department specialized in Huntington's disease? Any specific expertise in this area

This interview focuses on Huntington's disease, but do you also work with other patient groups?

- **Prompt questions:**
  ○ Do you work exclusively with people affected by Huntington's disease, or do you also work with other populations, such as those with dementia, Parkinson's, etc.?
  ○ Among those affected by Huntington's, do you work with people who have the disease themselves or with family members, such as those who carry the genetic risk, those at risk, or those who have been tested and found not to carry the gene?

When working with individuals who have Huntington's disease, at what stage of the disease are they typically? For instance, can they still function independently, manage their finances, handle household tasks, or are they entirely dependent on care or living independently?

- **[Clarify as much as possible about the target group and their functioning. If this includes multiple groups, the following questions may need to be asked for each specific group]**
  ○ Outpatient, extramural, intramural. Follow‐up and probe.

**Disclaimer:** For simplicity, I will refer to the people you work with as ‘patients’ for the remainder of the interview

What psychological issues or care requests do patients come to you with?

- **Prompt questions:**
  ○ What kinds of care requests do you frequently encounter in your daily practice?
  ○ Are these care requests specific to Huntington's disease?
  ○ Are these care requests specific to a particular stage of the disease?
  ○ How do you determine which interventions are suitable for a specific issue?

How do you conduct diagnostics and make a diagnosis? (Understanding the problem):

- **Prompt questions:**
  ○ Do you use an anamnesis?
  ○ Do you use questionnaires?
  ○ How do you understand the specific problem someone has?

How do you decide what kind of care to provide after making a diagnosis? (Setting an indication)

- **Prompt questions:**
  ○ What kind of care will we provide?
  ○ What therapeutic approaches do you use?
  ○ Which tools do you choose?

How do you determine which psychological interventions are suitable for which person and for which issue?

- **Prompt questions:**
  ○ Is the application of psychological interventions evidence‐based?
  ○ Is it specifically tailored to people with Huntington's disease?

Can you give your definition of what a psychological intervention is?

- **Prompt questions:**
  ○ This interview aims to inventory the interventions we use. How do you define a psychological intervention?
  ○ The toolbox analogy: which tools do you choose?

Can you describe the psychological interventions you use when working with people with Huntington's disease?

- **Prompt questions:**
  ○ In the previous question, you mentioned working with multiple groups. Do you use these interventions for all those groups? In which cases do you apply specific interventions?
  ○ Do these interventions belong to a specific psychological approach, or are they shared across multiple approaches? For example: Cognitive‐Behavioural Therapy (CBT), Mindfulness, Acceptance and Commitment Therapy (ACT), Interpersonal Therapy (IPT), Eye Movement Desensitization and Reprocessing (EMDR), Solution‐Focused Brief Therapy (SFBT), Family Therapy, Non‐directive Supportive Therapy, Psychoeducation, Relaxation Techniques, Reminiscence Therapy, or Mediation Therapy?
    **[Consider bringing a printed list to share during the interview]**
  ○ Regarding the care request, how is a specific problem addressed with this intervention?

Can you provide a specific example of a case where you used a psychological intervention and the outcome that was achieved?

- **Prompt questions:**
  ○ Can you share a success story?
  ○ Can you give an example of an intervention that was less successful? Why didn't it work?
    **[Implicit goal: to gather success stories and understand what doesn't work.]**

How do you determine whether your psychological interventions are effective for a particular care request?

- **Prompt questions:**
  ○ How do you measure the effectiveness of the psychological interventions you use?
  ○ Do you use a specific tool to measure effectiveness? Diagnostic tools?
  ○ Is the measurement tool the same for each intervention, or does it depend on factors such as the patient's stage of the disease, their personality, or the reason you chose the intervention?

Are there any challenges or obstacles you encounter in providing psychological care and treatment to people affected by Huntington's disease?

- **Prompt questions:**
  ○ What are those challenges, and how do they limit your ability to perform the psychological interventions?
  ○ How do you handle these challenges?

Are there any psychological interventions you would like to use when working with people with Huntington's disease but are currently unable to?

- **Prompt questions:**
  ○ Why not?
  ○ Are there interventions you would like to learn and apply to this group?
  ○ Are there interventions you currently don't have the opportunity to apply?
    **[Implicit goal: future research needs.]**

This conversation has covered a significant part of your work. Are there any other aspects you think might be important?

Thank you for sharing your knowledge and experience. Is there anything else you would like to add?

Once again, thank you very much for your time. The information you have provided will be invaluable to our research.

[End recording].
